# Supplementary material for: Human Induced Pluripotent Spheroids’ Growth Is Driven by Viscoelastic Properties and Macrostructure of 3D Hydrogel Environment
Source: Bioengineering (Basel). 2023 Dec 13;10(12):1418. doi: 10.3390/bioengineering10121418 (PMC10740696; doi:10.3390/bioengineering10121418)
Supplement: Supplementary file 1 [file bioengineering-10-01418-s001.zip › bioengineering-2744477-supplementary.pdf]

Supplementary Materials

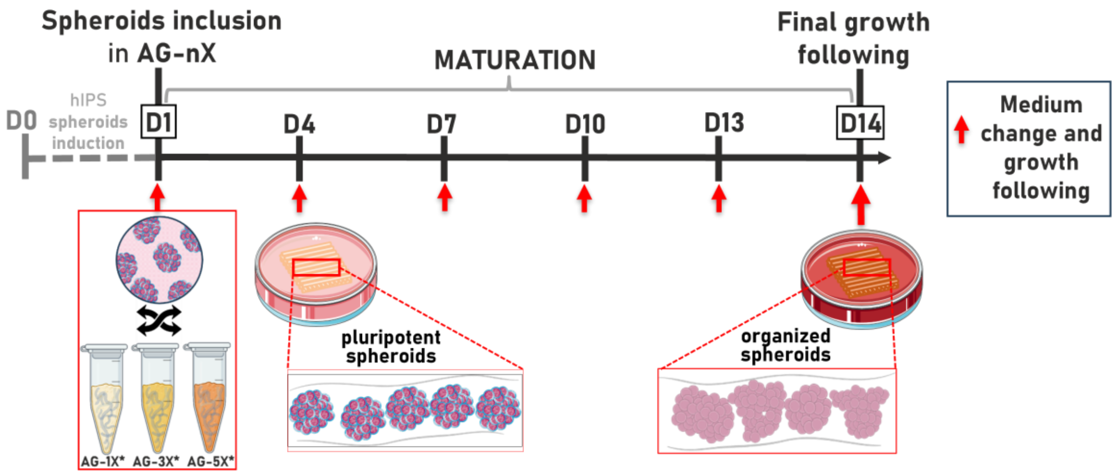

Figure S1. Study design, from the spheroids' induction to the final growth following.

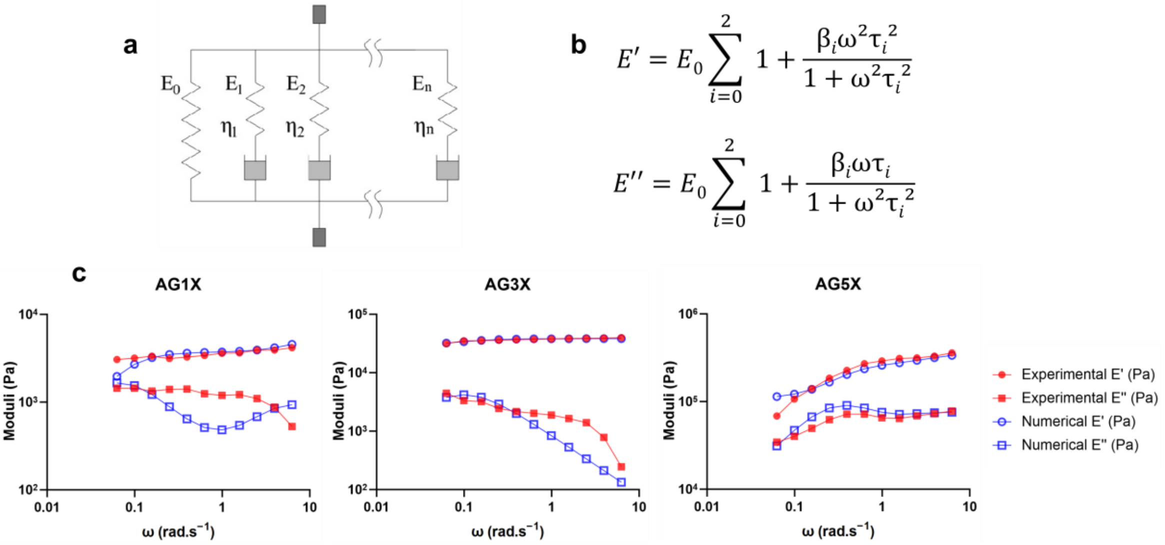

Figure S2. Fitting of a generalized second-order Maxwell model to experimentally measured modulus (E′ and E″) (**a**) Representation of measured data and calculated data with the 2nd-order Maxwell model. (**b**) Schematic representation of a generalized Maxwell model. (**c**) Equation of the E′ and E″ modules according to the 2nd-order Maxwell model.

Table S1. Parameters β,τ and E calculated for each AG-nX (n = 1, 3, 5). Results presented as mean ± SD (at least n = 3 per condition).

	β_1		τ_1	β_2		τ_2	E(kPA)	
	mean	SD	mean	mean	SD	mean	mean	SD
AG-1X	0.6	0.3	0.3	1.2	0.3	3.0	1.2	0.8
AG-3X	0.3	0.2	0.3	0.3	0.0	3.0	38.9	1.4
AG-5X	0.2	0.0	0.3	0.9	0.4	3.0	110.3	1.7
